# Response to Comments on: Guillain‐Barre Syndrome With Cytomegalovirus Infection After Allogeneic Hematopoietic Stem Cell Transplantation

**DOI:** 10.1002/kjm2.70263

**Published:** 2026-07-13

**Authors:** Chin‐Mu Hsu, Yong‐Jia Chen, Ju‐Jung Hou, Hui‐Hua Hsiao

**Affiliations:** ^1^ Division of Hematology‐Oncology, Department of Internal Medicine Kaohsiung Medical University Hospital Kaohsiung Taiwan; ^2^ Cancer Center Kaohsiung Medical University Hospital Kaohsiung Taiwan; ^3^ Department of Pharmacy Kaohsiung Medical University, Kaohsiung Medical University Kaohsiung Taiwan; ^4^ Faculty of Medicine Kaohsiung Medical University Kaohsiung Taiwan

Dear Editor,

1

We sincerely thank the reviewers for their comments on our case [1], which have allowed us to further consider our findings concerning the diagnosis of Guillain‐Barre syndrome (GBS). We appreciate the comments and our responses are as below.

Indeed, the diagnosis of GBS relies on clinical features, supplied by laboratory and electrophysiology findings [2]. The diagnosis is challenging and should rule out other possible etiologies. The European Academy of Neurology (ELN)/Peripheral Nerve Society (PNS) guideline of diagnosis requires three major features including progressive weakness of arms and legs, absent or decreased deep tendon reflex, and the progressive worsening for no more than 4 weeks combined with other features that support or defer the diagnosis [3]. We consider our patient to be a case of GBS as all three required features listed were fulfilled: symmetrically progressive weakness of arms and legs; decrease of deep tendon reflex; and the progression was less than 4 weeks. The 34‐day period between prodromal CMV infection and the onset of symptoms concurred with the guidelines supporting GBS with previous infection less than 6 weeks [4], while the worsening of progression was less than 4 weeks. Although the polyneuropathy from the NCV study raised some questions, two other NCV studies, 1 week apart, revealed the same results with delayed F‐wave, which support GBS. The patient is likely to have the sensory‐motor form of GBS, which showed mild sensory symptoms. CSF studies also supported the diagnosis with scanty cells, high protein level, and high IgG level; additionally, the patient had bilateral facial palsy, autonomic dysfunction, and limbs pain, all of which support the diagnosis. Regretfully, we lacked specific antibodies and MRI evidence; however, the diagnosis was determined based on the features described above.

Surely, other possible etiologies should be ruled out. The patient had no fever at the onset, and the studies of CSF, including acid‐fast stain, cryptococcus stain, micro‐organism studies and cultures, were all negative, while serum levels of Vitamin B12 and folic acid were normal. Though GBS might also come from the disease itself and previous toxic chemotherapy agents, bone marrow examination revealed complete remission negative for MRD after transplantation and the patient did not suffer from neuropathy after therapy till this time, thereby ruling out these etiologies.

We concur that IVIG treatment might help the patient with GBS [2, 3], although the patient in this case refused IVIG infusion being fearful of possible side effects, especially anaphylaxis; alternatively, plasma exchange plus steroid therapy was administered (as the standard of care for this severe disease), resulting in later improvement.

GBS is a potentially devastating, life‐threatening yet treatable disease; accordingly, diagnosis and prompt treatment strategy are crucial. Though rare [4], we report this case for the interest of GBS, especially post‐CMV infection after allogeneic transplantation.

## Conflicts of Interest

The authors declare no conflicts of interest.

## Data Availability

The data that support the findings of this study are available from the corresponding author upon reasonable request.
